# SS-31 improves post-cardiac arrest brain injury by inhibiting microglial ferroptosis and polarization

**DOI:** 10.1016/j.neurot.2025.e00772

**Published:** 2025-10-24

**Authors:** Tangxing Jiang, Huidan Zhang, Yijun Sun, Xianfei Ji, Li Xue, Chang Pan, Yunyun Guo, Feng Xu

**Affiliations:** a Department of Emergency Medicine, Qilu Hospital of Shandong University, Jinan, China; b Shandong Provincial Clinical Research Center for Emergency and Critical Care Medicine, Institute of Emergency and Critical Care Medicine of Shandong University, Chest Pain Center, Qilu Hospital of Shandong University, Jinan, China; c Medical and Pharmaceutical Basic Research Innovation Center of Emergency and Critical Care Medicine, China's Ministry of Education, Shandong Provincial Engineering Laboratory for Emergency and Critical Care Medicine, Key Laboratory of Emergency and Critical Care Medicine of Shandong Province, Key Laboratory of Cardiopulmonary-Cerebral Resuscitation Research of Shandong Province, Qilu Hospital of Shandong University, Jinan, China; d NMPA Key Laboratory for Clinical Research and Evaluation of Innovative Drug, Qilu Hospital of Shandong University, Jinan, China; e National Key Laboratory for Innovation and Transformation of Luobing Theory, The Key Laboratory of Cardiovascular Remodeling and Function Research, Chinese Ministry of Education, Chinese National Health Commission and Chinese Academy of Medical Sciences, Qilu Hospital of Shandong University, Jinan, China; f Department of Critical Care Medicine, Shenzhen Second People's Hospital, The First Affiliated Hospital of Shenzhen University, Shenzhen, China

**Keywords:** Cardiac arrest, SS-31, Microglial polarization, Ferroptosis, Sesn2

## Abstract

Accumulating evidence suggests that ferroptosis and mitochondrial dysfunction contribute significantly to brain injury following cardiac arrest (CA) and resuscitation. SS-31, a novel mitochondria-targeting peptide, has demonstrated protective effects against mitochondrial dysfunction induced by ischemia/reperfusion injury. This study aimed to investigate the neuroprotective effects of SS-31 in post-CA brain injury and clarify the underlying signaling mechanisms. An in vivo rat model of CA and resuscitation was established. Following resuscitation, animals were randomly divided into three groups: a saline-treated control group, an SS-31-treated group, and a sham-operated control group. Survival rates, neurological deficit scores, serum neuronal injury markers (NSE and S100B), and histopathological changes were evaluated for up to 72 ​h post-resuscitation. Mechanistically, ferroptosis-related signaling pathways were examined, including glutathione peroxidase 4 (GPX4) expression, iron accumulation, oxidative stress markers, and pro-inflammatory cytokine levels, utilizing microglia-specific Sesn2 knockdown via adeno-associated virus vectors. *In vitro* experiments were performed on BV2 cells subjected to oxygen-glucose deprivation/reoxygenation, assessing cell viability, lipid peroxidation, ferroptosis-associated protein expression, and cytokine secretion following SS-31 intervention. Brain injury post-CA and resuscitation is significantly accompanied by ferroptosis of microglia. Treatment with SS-31 substantially improved survival rates, reduced neurological deficits, and lowered serum NSE and S100B levels. Mechanistically, SS-31 attenuated ferroptosis and promoted an anti-inflammatory shift in microglial polarization by enhancing GPX4 expression and decreasing iron content, oxidative stress, and pro-inflammatory cytokines. These effects were primarily mediated via the Sesn2 signaling pathway. SS-31 could effectively improve post-CA brain injury, in which the mechanism was potentially related to the inhibition of microglial ferroptosis and polarization through the regulation of Sesn2 signaling pathway.

## Highlights

• SS-31 enhances survival and neurological outcomes after cardiac arrest.
• SS-31 reduces microglial ferroptosis and promotes a shift from the pro-inflammatory M1 phenotype toward the anti-inflammatory M2 phenotype.
• SS-31 exerts neuroprotection against post-cardiac arrest brain injury by regulating Sesn2-mediated signaling.

## Graphical abstract

**Figure:**
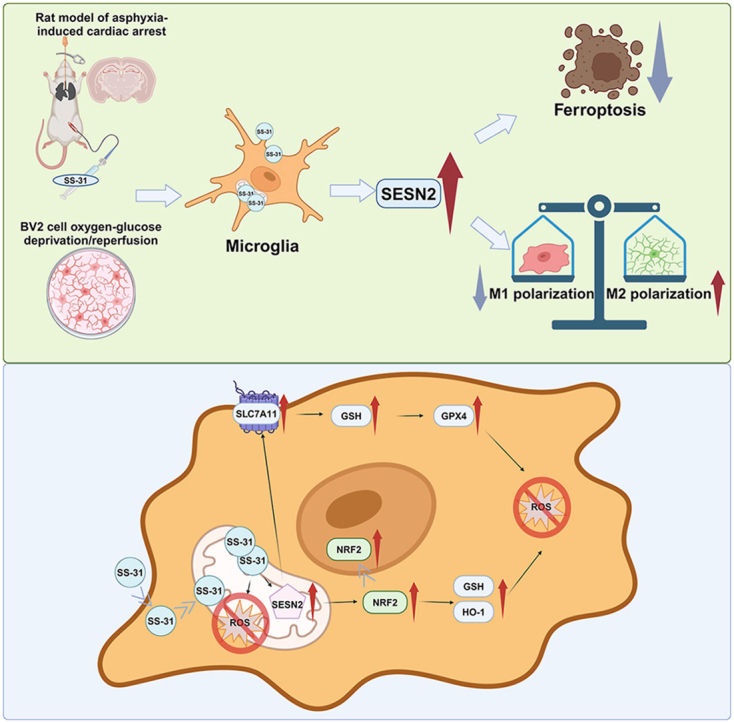

## Introduction

Out-of-hospital cardiac arrest (OHCA) poses a significant global public health challenge, with the annual incidence of OHCA treated by emergency medical services ranging from 28 to 244 cases per 100,000 people worldwide [1]. Cardiac arrest (CA) induces global brain ischemia, leading to severe brain injury. Even with successful initial resuscitation, up to 70 ​% of patients admitted to the hospital ultimately die due to brain injury resulting from the CA [2].

Neuroinflammation is a key mechanism of injury in brain damage following CA. This inflammatory response is driven by the resident macrophage-like cells of the central nervous system, known as microglia [3]. Microglia play a crucial role in the brain's immunopathological response after CA [4]. Once activated, microglia can adopt different phenotypes, generally categorized into two main types based on their function: the pro-inflammatory M1 phenotype and the anti-inflammatory M2 phenotype. Studies have shown that microglia in areas of cerebral ischemia-reperfusion injury predominantly display the M1 phenotype, creating a pro-inflammatory microenvironment that contributes to neuronal damage [5,6]. Therefore, reducing the number of M1 microglia is a potential therapeutic target for alleviating neuroinflammation and ultimately improving brain function after CA.

Iron is one of the most abundant metals in the brain, and microglia are among the cells with the highest iron storage capacity in the brain. Dysregulation of iron homeostasis in microglia has been linked to the progression of several neurodegenerative diseases, such as Parkinson's disease [7]. Moreover, microglia may be particularly susceptible to ferroptosis, a form of iron-dependent cell death. Unlike apoptosis and necroptosis, ferroptosis is driven by iron-dependent lipid peroxidation and is characterized by three key features: inactivation of Glutathione Peroxidase 4 (GPX4), iron accumulation, and oxidation of polyunsaturated fatty acids [8,9]. Furthermore, iron accumulation can trigger microglial activation and the release of pro-inflammatory cytokines, such as tumor necrosis factor-alpha (TNF-α), interleukin-1β (IL-1β), and interleukin-6 (IL-6) [[10], [11], [12]].

Currently, there are very few therapies available to improve neurological outcomes after CA. The only promising option is mild therapeutic hypothermia, but even with this treatment, the optimal target temperature remains unclear, and there is no strong consensus on its effectiveness [13]. Therefore, there is an urgent need to explore effective neuroprotective strategies for CA patients. Ischemia-reperfusion injury caused by CA significantly impacts brain mitochondrial function, playing a critical role in post-CA brain injury and making it a key therapeutic target [14]. SS-31 is a novel mitochondria-targeting protective peptide that not only binds to cardiolipin on the inner mitochondrial membrane but also inhibits the peroxidase activity of cytochrome *c*, reducing electron leakage [15]. This mechanism decreases mitochondrial reactive oxygen species (ROS) production, suppressing oxidative stress and lipid peroxidation while improving mitochondrial bioenergetics. Notably, lipid peroxidation and ROS accumulation are also the key features of ferroptosis and form the pathophysiological basis of ischemia-reperfusion injury. Previous studies have shown that SS-31 can attenuate ischemic brain injury by maintaining redox balance [16,17]. Additionally, SS-31 has also shown therapeutic potential in alleviating neuroinflammatory damage [18].

Therefore, in this study, we hypothesize that SS-31 administration may improve neurological dysfunction in rats after CA by mitigating microglial ferroptosis and reducing the secretion of inflammatory cytokines from M1-polarized microglia.

## Materials and methods

### Asphyxia-induced CA model establishment

Male Sprague–Dawley rats (230–300 ​g) were obtained from the Laboratory Animal Center of Qilu Hospital, Shandong University, Jinan, China. The rats were housed in a controlled environment on a 12-h light–dark cycle and provided with a standard laboratory diet. For the experiments, the animals were fasted overnight with free access to water. A previously established rat model of CA was employed [19,20]. Briefly, the rats were anesthetized using 5 ​% isoflurane in room air (21 ​% oxygen) within a plastic induction chamber. Once fully anesthetized (no pain response, absence of corneal reflex), the trachea was orally intubated. The rats were mechanically ventilated and maintained under 2 ​% isoflurane anesthesia. A PE-50 catheter was inserted through the left femoral artery into the aorta to measure mean aortic pressure (MAP). Additionally, a microcatheter was placed in the left femoral vein for drug administration, and a continuous lead II electrocardiogram was recorded. Pancuronium bromide (2 ​mg/kg) was administered intravenously to achieve full muscle relaxation. After administering pancuronium bromide, the rats received 0.5 ​% isoflurane in room air (21 ​% oxygen) for 5 ​min. CA was induced by asphyxia by turning off the ventilator and clamping the endotracheal tube, leading to a rapid decline in hypotension and heart rate. CA was defined as a MAP of <20 ​mmHg. After 8 ​min of asphyxia, chest compressions were performed at a rate of 200 beats/min using a pneumatically driven mechanical chest compressor. Mechanical ventilation with 100 ​% oxygen and a bolus injection of 10 ​μg/kg epinephrine through the venous line were started 30 ​s before chest compressions. Compressions and epinephrine injections were repeated every 5 ​min until return of spontaneous circulation (ROSC) was achieved. ROSC was defined as the return of supraventricular rhythm with MAP >50 ​mmHg sustained for at least 5 ​min. Only rats that achieved ROSC within 10 ​min were included in subsequent studies. This criterion ensured consistent CA duration and reduced variability. Following ROSC, the rats were weaned off the ventilator, extubated, and returned to their cages with free access to food and water.

### Experimental design

**Fig. 1:**
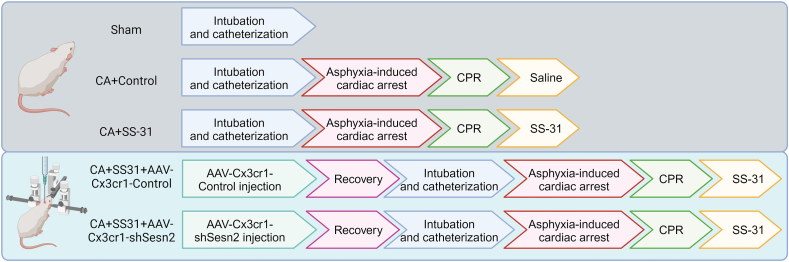
**Experimental design**.

The study consisted of two parts (Fig. 1). In the first part, rats were randomly assigned to one of three groups: the Sham group, the CA ​+ ​Control group (receiving saline after successful resuscitation), and the CA ​+ ​SS-31 group (receiving SS-31 after successful resuscitation). The second part focused on mechanism exploration. Rats were randomly assigned to receive a brain injection of either AAV-Cx3cr1-Control or Cx3cr1-shSesn2 20 days prior to CA induction. Following successful ROSC, these rats were treated with SS-31, administered intravenously at 30 ​mg/kg, diluted in saline (#S9803, Selleck, USA), following previously established protocols with slight modifications [21]. Rats in the Sham group underwent all procedures except for CA. Each group initially included 17 rats, with 7 rats euthanized 24 ​h post-CA for hippocampal tissue and blood collection. The remaining 10 rats were observed for 72-h survival and neurological function assessment.

### Survival analysis and neurologic deficit score

For the 72-h survival study, all catheters were removed, and surgical wounds were sutured 6 ​h post-CA. Rats were monitored every 12 ​h. The Neurologic Deficit Score (NDS), which ranged from 0 (death or brain death) to 80 (no neurologic deficits), was used to assess arousal level, cranial nerve reflexes, motor function, and simple behavioral responses [22]. Two independent, blinded researchers evaluated the NDS at 24, 48, and 72 ​h post-CA for all groups. A detailed scoring system for NDS (0–80 points) is provided in Supplementary Table 1.

### Adeno-associated virus injection

Rats were anesthetized with intraperitoneal sodium pentobarbital and secured in a stereotaxic frame (RWD Life Science, China). A microglia-specific adeno-associated virus (AAV) under the Cx3cr1 promoter was used to achieve specific knockdown of Sesn2 in microglia. Using a 10 ​μL microsyringe (Gaoge, China), 2.0 ​μL of AAV-Cx3cr1-Control or AAV-Cx3cr1-shSesn2 (AAV2/9, Hanheng, China) was bilaterally injected into the hippocampus at a rate of 0.2 ​μL/min (coordinates from the bregma: −3.48 ​mm; medial/lateral: ±1.8 ​mm; dorsal/ventral: −2.55 ​mm). Stereotaxic coordinates for hippocampus were guided by Paxinos & Watson, The Rat Brain in Stereotaxic Coordinates (6th edition) and cross-checked with coordinates reported by Song et al., 2018 (established for Wistar rats at 8 weeks of age) [23]. The needle remained in place for 10 ​min post-injection to facilitate viral diffusion and prevent backflow. Afterward, the needle was slowly withdrawn, bone wax was applied to seal the craniotomy, and the incision was sutured. A 20-day recovery period was allowed for sufficient viral expression before CA induction. The shSesn2 sequence used was 5′-AGCCTACAGCCTCACCTATAA-3'.

### Cell culture

BV2 mouse microglia cells (iCell Biosciences, China) were cultured in high-glucose DMEM (Sigma-Aldrich, USA) supplemented with 10 ​% fetal bovine serum (FBS) and 1 ​% penicillin-streptomycin. Cells were maintained in T75 flasks at 37 ​°C in a humidified atmosphere containing 5 ​% CO2 when not in use for experiments.

### Oxygen-glucose deprivation and reoxygenation

The culture medium was replaced with glucose-free DMEM. Cells were placed in a 37 ​°C incubator with a humidified atmosphere of 1 ​% O2, 5 ​% CO2, and 94 ​% N2 for oxygen deprivation. After the deprivation period, cells were transferred to normoxic conditions and incubated in high-glucose DMEM with 10 ​% FBS to terminate oxygen-glucose deprivation. The medium was supplemented with SS-31 for further treatment.

### RNA interference

Sesn2 expression was inhibited using short hairpin RNA (shRNA). shSesn2 and its negative control (shNC) were purchased from Biosune Corporation (Shanghai, China). BV2 cells were transfected with siRNA using RNA iMAX reagent (L3000015, Invitrogen, USA) according to the manufacturer's protocol. The shSesn2 sequence was 5′-AGCCTGCAGCCTCACCTATAA-3′.

### CCK-8 assay

Cell viability was assessed using the Cell Counting Kit-8 (Solarbio, China) according to the manufacturer's instructions. BV2 cells were seeded in 96-well plates. After the specified treatments, CCK-8 solution was added to each well, and the plates were incubated for the recommended times. Cell viability was measured by determining the optical density (OD) at 450 ​nm.

### Lipid peroxidation detection

Cells were incubated with fresh medium containing 5 ​μmol/L C11-BODIPY 581/591 (D3861, ThermoFisher, USA) at 37 ​°C for 30 ​min in the dark. After incubation, the cells were washed with PBS, stained with Hoechst for nuclear visualization, and imaged. Lipid peroxidation, indicated by the oxidation of the cell membrane by lipid hydroperoxides, results in a shift of the fluorescence emission peak from 590 ​nm (red) to 510 ​nm (green). The green/red fluorescence ratio was used to assess oxidative damage to the cell membrane.

### Iron, MDA, GSH, and SOD assays

Tissue iron levels were measured using an Iron Assay Kit (A039-2, Jiancheng Biotechnology, China) according to the manufacturer's instructions. Cellular Fe^2+^ levels were assessed using an Iron Assay Kit (ab83366, Abcam, UK) following the provided protocol. MDA levels were measured using an MDA Detection Kit (A003-1, Jiancheng Biotechnology, China). SOD level was determined using an SOD Assay Kit (A001-1, Jiancheng Biotechnology, China), and GSH levels were measured using a GSH Assay Kit (A006-2, Jiancheng Biotechnology, Nanjing, China).

### Enzyme-linked immunosorbent assay (ELISA)

Levels of IL-1β, IL-6, and TNF-α in hippocampal tissue were measured using rat ELISA kits (Boster Biological Technology, China) following the manufacturer's instructions. Briefly, rat hippocampal tissue was lysed and homogenized, and the homogenate was centrifuged at 12,000 ​rpm for 10 ​min at 4 ​°C. The supernatant was collected for ELISA. In subsequent cell experiments, IL-1β, IL-6, and TNF-α levels in the supernatant of BV2 cell culture were analyzed using a similar ELISA protocol. Additionally, rat S100B and neuron-specific enolase (NSE) concentrations were measured using S100B and NSE ELISA kits (Cloud-Clone, China).

### Western blot

Tissue or cell lysates were prepared using RIPA buffer containing protease and phosphatase inhibitors. Protein concentrations were quantified using a BCA protein assay kit. Protein samples were separated by SDS-PAGE and transferred to PVDF membranes, which were blocked with 5 ​% skim milk and incubated overnight at 4 ​°C with primary antibodies (NRF2, 1:1000, 16396-1-AP; COX2, 1:1000, 12375-1-AP; SLC7A11, 1:1000, 26864-1-AP; HO-1, 1:1000, 10701-1-AP; GPX4, 1:1000, 67763-1-Ig; Sesn2, 1:1000, 10795-1-AP; CD206, 1:1000, 18704-1-AP; INOS, 1:1000, 18985-1-AP; β-actin, 1:1000, 66009-1-Ig; all from Proteintech, China). The membranes were then incubated with goat anti-mouse or goat anti-rabbit secondary antibodies (Proteintech, China) for 2 ​h at room temperature. The protein bands were visualized using a chemiluminescence detection system, and results were normalized to β-actin levels.

### Immunofluorescence staining

Rat brains were quickly removed after perfusion and immersed in 4 ​% paraformaldehyde at 4 ​°C for 24 ​h. They were then transferred sequentially into 20 ​% and 30 ​% sucrose solutions for dehydration until the tissues sank to the bottom. Brains were cryosectioned at 10 ​μm using a cryostat, and sections were collected and mounted. The sections were washed three times with PBS. For cultured cells, fixation was performed using 4 ​% paraformaldehyde for 30 ​min at room temperature, followed by three washes with PBS.

Both tissue sections and cultured cells were blocked with 10 ​% goat serum containing 0.1 ​% Triton X-100 for 1 ​h at room temperature. Primary antibodies (Iba1, 1:100, ab178846, Abcam, UK; GPX4, 1:50, sc-166570, Santa Cruz, US; CD206, 1:50, 60143-1-Ig, Proteintech, China; iNOS, 1:1000, 18985-1-AP, Proteintech, China; SLC7A11, 1:100, 26864-1-AP, Proteintech, China) were applied and incubated overnight at 4 ​°C. Secondary antibodies were applied for 2 ​h at room temperature, followed by DAPI (ab104139, Abcam, UK) for nuclear staining. Images were captured using a fluorescence or confocal microscope, and six random views from each group were analyzed.

### HE and Nissl staining, and Prussian Blue staining

HE Staining: Sections were stained with hematoxylin for 3 ​min and placed in 1 ​% acidic alcohol for 5 ​min. After thorough rinsing with tap water to achieve a blue hue. Nissl Staining: Sections were stained with toluidine blue solution (G3668; Solarbio, China), followed by 0.5 ​% eosin for 3 ​s and rinsing with running water. Prussian Blue Staining: Prussian blue staining was performed using a staining kit (G1029, Servicebio, China). Sections were stained with a mixture of Prussian Blue Solution A and B for 1 ​h and washed twice with distilled water. They were then stained with Prussian Blue Solution C for 3 ​min and rinsed with running water.

### ROS staining

Sections were incubated in physiological saline containing 10 ​μmol/L dihydroethidium (DHE; Sigma–Aldrich, USA) for 30 ​min at 37 ​°C in the dark and then washed twice with PBS. DAPI (ab104139, Abcam, UK) was applied for nuclear staining.

### Transmission electron microscopy (TEM)

Samples stored in 2.5 ​% glutaraldehyde were further fixed with 1 ​% osmium tetroxide, dehydrated, and embedded in epoxy resin. Using standard three-dimensional localization, 100 ​nm sections were cut and mounted on copper grids. Sections were double-stained with lead citrate and uranyl acetate. Mitochondrial morphology was observed and recorded using a Hitachi H-7650 TEM (Shiga, Japan).

### Statistical analysis

Continuous variables are presented as mean ​± ​SD after confirming normal distribution using the Kolmogorov-Smirnov test. Group comparisons were conducted using one-way or two-way ANOVA followed by Tukey's post hoc test. Neurological Deficit Scores were analyzed with non-parametric tests. Survival analysis was performed using Kaplan-Meier plots and log-rank tests. A p-value of <0.05 was considered statistically significant. All statistical analyses were conducted using GraphPad Prism 10.0 (GraphPad Software, USA).

## Results

### Baseline characteristics of rats and resuscitation characteristics

A total of 94 rats were used in this study, of which 85 achieved ROSC within 10 ​min and were included in the analysis. Two rats died during anesthesia induction prior to CA, and 7 rats were euthanized because they failed to regain supraventricular rhythm after 5 ​min of CPR or could not maintain a MAP of over 50 ​mmHg for 5 ​min within 10 ​min of resuscitation.

No significant differences were observed between the groups in baseline body weight, baseline heart rate, baseline mean arterial pressure, time from asphyxia to CA, or CPR duration needed to achieve ROSC (Table 1).

**Table 1:** Baseline characteristics of rats and resuscitation characteristics.

Variable	Sham	CA ​+ ​Control	CA ​+ ​SS-31	CA ​+ ​SS31+AAV-Cx3cr1-Control	CA ​+ ​SS31+AAV-Cx3cr1-shSesn2
Body weight (g)	269.5 ​± ​14.91	279.7 ​± ​10.09	274.3 ​± ​17.77	268.3 ​± ​18.21	266.7 ​± ​23.12
Heart rates (beats/min)	332 ​± ​33.77	349.4 ​± ​45.22	353.6 ​± ​27.25	347.5 ​± ​25	344.4 ​± ​47.01
Mean arterial pressure (mmHg)	105.6 ​± ​10.21	110.3 ​± ​13.5	105 ​± ​6.374	110.5 ​± ​9.709	113 ​± ​11.4
Time from asphyxia to cardiac arrest (s)	N/A	263.4 ​± ​32.86	273.8 ​± ​37.88	270.1 ​± ​38.95	267.8 ​± ​35.16
CPR time to achieve ROSC (s)	N/A	47.06 ​± ​11.49	45.94 ​± ​10.01	40.82 ​± ​11.26	42.41 ​± ​9.689

Values are expressed as mean ​± ​SD. CPR: Cardiopulmonary resuscitation; N/A: Not applicable; ROSC: Return of spontaneous circulation.

### SS-31 improves survival rate and neurological outcomes in rats after CA

**Fig. 2:**
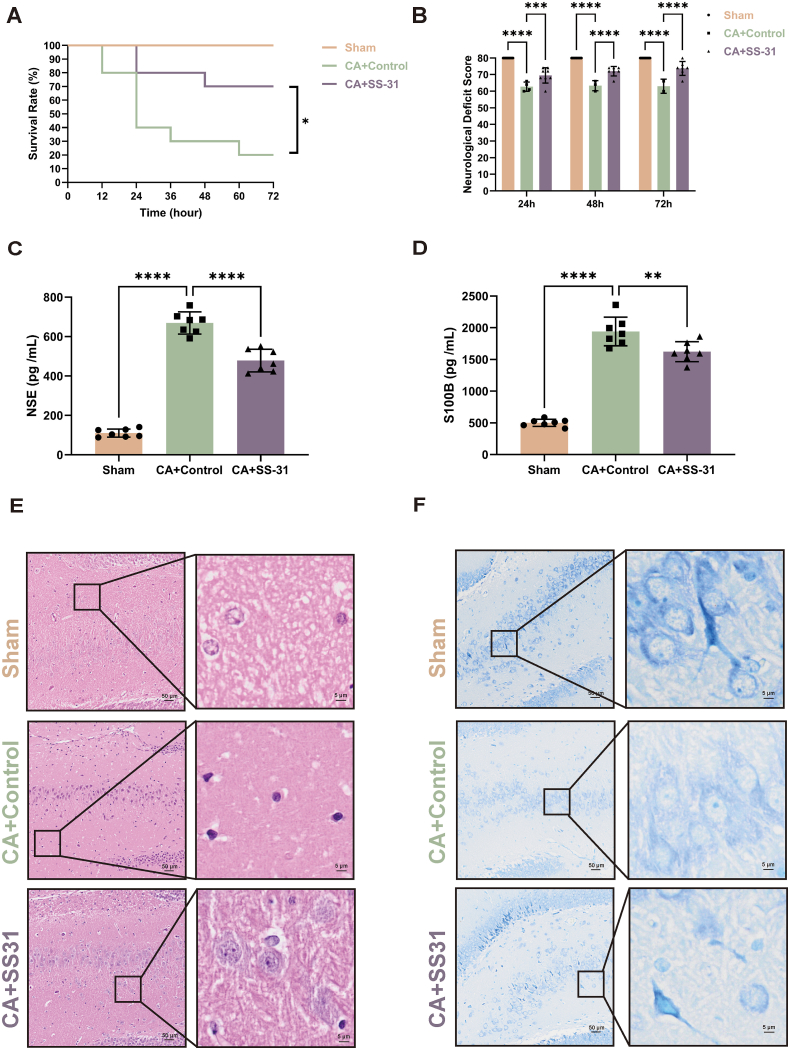
**SS-31 Improves Survival Rate and Neurological Outcomes in Rats After Cardiac Arrest**. (A) Survival analysis across groups. (B) Neurological deficit scores at 24, 48, and 72 ​h after ROSC. (C) Serum levels of neuron-specific enolase (NSE). (D) Serum levels of S100B. (E) Hematoxylin and eosin (H&E) staining of the hippocampus. (F) Nissl staining of the hippocampus. Statistical significance is indicated as ∗∗P ​< ​0.01, ∗∗∗P ​< ​0.001, ∗∗∗∗P ​< ​0.0001. Scale bars are shown in individual panels.

Rats were randomly assigned to three groups: Sham, CA ​+ ​Control, and CA ​+ ​SS-31, to evaluate the efficacy of SS-31 following cardiac arrest. No mortality was observed in the Sham group during the observation period. At 72 ​h post-CA, the CA ​+ ​SS-31 group demonstrated a significantly higher survival rate compared to the CA ​+ ​Control group (Fig. 2A). Neurological function was assessed at 24, 48, and 72 ​h post-CA using a neurological deficit score (Fig. 2B). The CA ​+ ​SS-31 group showed significant improvement at all time points compared to the CA ​+ ​Control group.

Serum levels of neuronal injury markers, including NSE (Fig. 2C) and S100B (Fig. 2D), were significantly elevated in the CA ​+ ​Control group compared to the Sham group but were notably reduced in the CA ​+ ​SS-31 group. Histological analysis using H&E staining revealed extensive neuronal damage in the CA ​+ ​Control group, characterized by necrotic cells and nuclear deformation. In contrast, the CA ​+ ​SS-31 group showed significantly less neuronal damage and improved cellular morphology, with more intact cells (Fig. 2E). Further assessment of neuronal damage using Nissl staining showed a large number of light-blue Nissl bodies in the cytoplasm of neurons in the Sham group. Both the CA ​+ ​SS-31 and CA ​+ ​Control groups exhibited fewer Nissl bodies compared to the Sham group. However, the CA ​+ ​SS-31 group retained more Nissl bodies than the CA ​+ ​Control group, indicating a neuroprotective effect of SS-31(Fig. 2F). These results demonstrate that post-CA treatment with SS-31 significantly improves survival rates and neurological function while reducing neuropathological damage.

### SS-31 reduces ferroptosis in hippocampus after CA

**Fig. 3:**
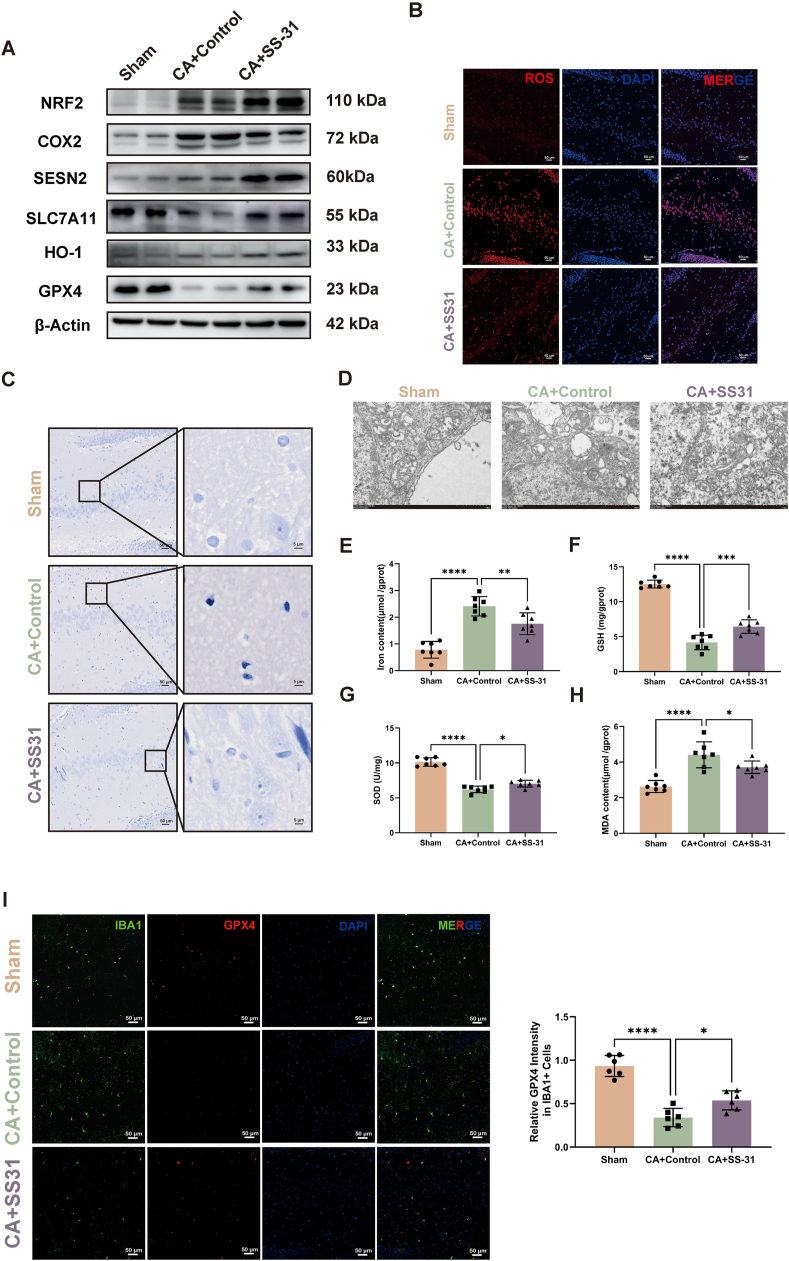
**SS-31 Reduces Ferroptosis in the Hippocampus After Cardiac Arrest**. (A) Western blot analysis of ferroptosis-related proteins (GPX4, SLC7A11, HO-1, NRF2, COX2, SESN2) in the hippocampus. (B) DHE staining showing ROS production in the hippocampus. (B) DHE staining images showing ROS production in the hippocampus. (C) Prussian blue staining indicating iron accumulation in the hippocampus. (D) Electron microscopy images of hippocampal mitochondria. (E) Iron content in the hippocampus. (F) GSH levels in the hippocampus. (G) SOD levels in the hippocampus. (H) MDA content in the hippocampus. (I) Immunofluorescence staining of GPX4 (red) in IBA1+ microglia (green) with DAPI (blue) nuclear staining. The bar graph shows quantitative analysis of relative GPX4 intensity in IBA1+ cells. Statistical significance is indicated as ∗P ​< ​0.05, ∗∗P ​< ​0.01, ∗∗∗P ​< ​0.001, and ∗∗∗∗P ​< ​0.0001. Scale bars are shown in individual panels.

Ferroptosis is a form of cell death driven by iron-dependent lipid peroxidation, characterized by three key indicators: decreased GPX4, iron accumulation, and lipid peroxidation. To investigate the association between post-CA brain injury and ferroptosis, as well as the therapeutic efficacy of SS-31, we conducted the following analyses: Western blot analysis demonstrated that SS-31 treatment increased the expression of ferroptosis-protective proteins GPX4, SLC7A11, SESN2, HO-1, and NRF2 in the CA ​+ ​SS-31 group compared to the CA ​+ ​Control group (Fig. 3A, Supplementary Fig. 2A). In contrast, levels of COX2, a marker of oxidative stress, were reduced in the CA ​+ ​SS-31 group. DHE staining confirmed a significant reduction in ROS production in the CA ​+ ​SS-31 group (Fig. 3B, Supplementary Fig. 2B). Prussian blue staining indicated decreased iron accumulation in the CA ​+ ​SS-31 group compared to the CA ​+ ​Control group (Fig. 3C). Additionally, electron microscopy revealed less mitochondrial damage, including reduced mitochondrial shrinkage, preserved cristae, and an intact outer membrane, in the CA ​+ ​SS-31 group (Fig. 3D). SS-31 treatment also reduced iron content (Fig. 3E) and lipid peroxidation marker MDA (Fig. 3H), while increasing antioxidant markers such as GSH and SOD (Fig. 3F and G), suggesting enhanced resistance to ferroptosis. Given that microglia are the primary immune cells in the central nervous system and play a crucial role in neuroinflammation after CA, we focused our investigation on ferroptosis within microglia. Immunofluorescence staining revealed inactivation of GPX4 in microglia following CA, which was restored with SS-31 treatment (Fig. 3I). These findings indicate that SS-31 mitigates ferroptosis in the hippocampus following CA, with microglia being one of the key affected cell types.

### SS-31 reduces ferroptosis induced by hypoxia-reoxygenation in BV2 cells

To simulate the response of hippocampal microglia following CA, we used a BV2 microglial cell line and an oxygen-glucose deprivation/reoxygenation (OGD/R) model. Cell viability of BV2 cells was assessed using a CCK-8 assay after different durations of oxygen-glucose deprivation followed by 2 ​h of reoxygenation (OGD 1 ​h/R 2h, OGD 2 ​h/R 2h, OGD 3 ​h/R 2h, OGD 4 ​h/R 2h). Results demonstrated a significant decrease in cell viability with increasing OGD duration. No significant difference was found between OGD 3 ​h/R 2h and OGD 4 ​h/R 2h, so OGD 3 ​h/R 2h was selected as the standard condition (Supplementary Fig. 1A). To determine the optimal SS-31 concentration, BV2 cells were subjected to OGD for 2 ​h followed by 2 ​h of reoxygenation, during which they were treated with SS-31 at concentrations of 1, 2.5, 5, 10, and 20 ​μM. The results indicated that SS-31 significantly improved cell viability at 10 ​μM and 20 ​μM. However, no additional benefit was observed at concentrations higher than 10 ​μM, so 10 ​μM was chosen as the treatment concentration (Supplementary Fig. 1B).

**Fig. 4:**
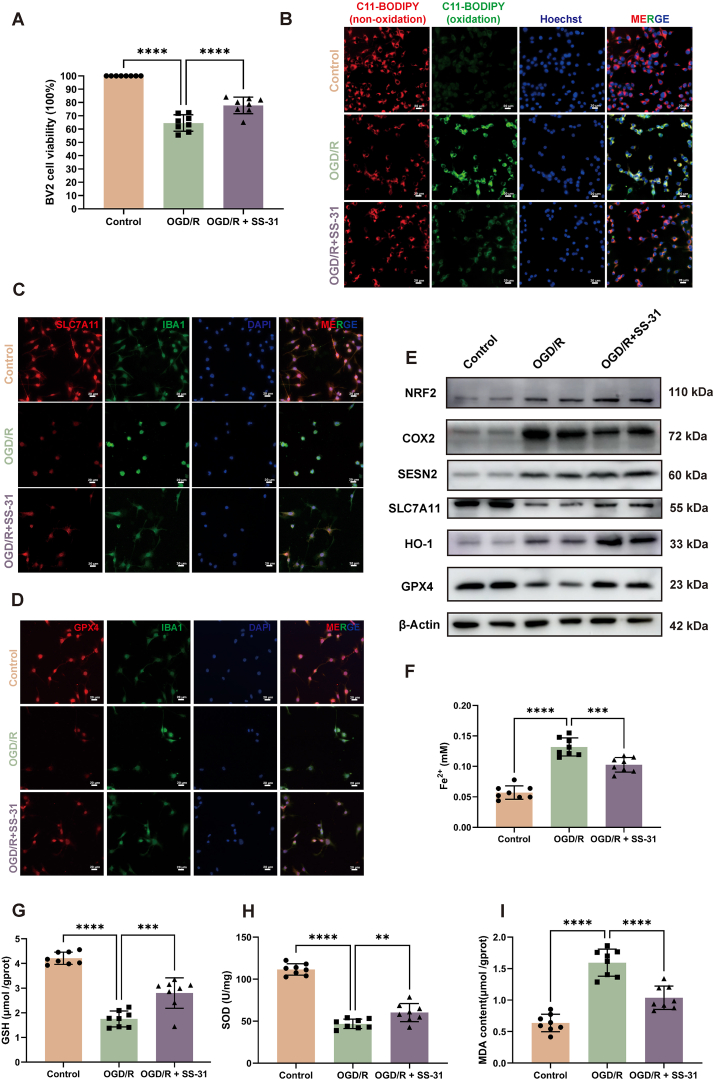
**SS-31 Reduces Ferroptosis Induced by Hypoxia-Reoxygenation in BV2 Cells**. (A) Cell viability assay (B) Lipid peroxidation in BV2 cells, as indicated by C11-BODIPY staining (non-oxidized, red; oxidized, green) and nuclear staining with Hoechst (blue). (C) Immunofluorescence staining for SLC7A11 (red), IBA1 (green), and DAPI (blue) in BV2 cells. (D) Immunofluorescence staining for GPX4 (red), IBA1 (green), and DAPI (blue) in BV2 cells. (E) Western blot analysis of ferroptosis-related proteins (GPX4, SLC7A11, HO-1, NRF2, COX2, Sesn2) in BV2 cells. (F) Fe^2+^ levels measured in BV2 cells. (G) GSH levels in BV2 cells. (H) SOD levels in BV2 cells. (I) MDA content in BV2 cells. Statistical significance is represented as ∗∗P ​< ​0.01, ∗∗∗P ​< ​0.001, and ∗∗∗∗P ​< ​0.0001. Scale bars are shown in individual panels.

Cell viability significantly decreased following OGD/R treatment compared to the control but was partially restored in the OGD/R ​+ ​SS-31 group (Fig. 4A). Lipid peroxidation, as measured by C11-BODIPY staining, was reduced in the OGD/R ​+ ​SS-31 group, suggesting decreased oxidative membrane damage (Fig. 4B, Supplementary Fig. 3A). Immunofluorescence staining showed that SS-31 treatment restored the expression of anti-ferroptotic proteins SLC7A11 and GPX4, both of which were reduced after OGD/R alone (Fig. 4C and D, Supplementary Fig. 3B and 3C). Western blot analysis further demonstrated that SS-31 increased the levels of NRF2, SLC7A11, GPX4, SESN2 and HO-1 while reducing COX2 expression under OGD/R conditions, indicating reduced oxidative stress and ferroptosis (Fig. 4E, Supplementary Fig. 3D). Additionally, SS-31 decreased iron content and the lipid peroxidation marker MDA, while increasing antioxidant markers GSH and SOD levels, further supporting its anti-ferroptotic effects (Fig. 4F–I). Our in vitro study validated the therapeutic effect of SS-31 in reducing microglial ferroptosis.

### SS-31 promotes microglial M1 to M2 polarization after CA and hypoxia/reoxygenation

**Fig. 5:**
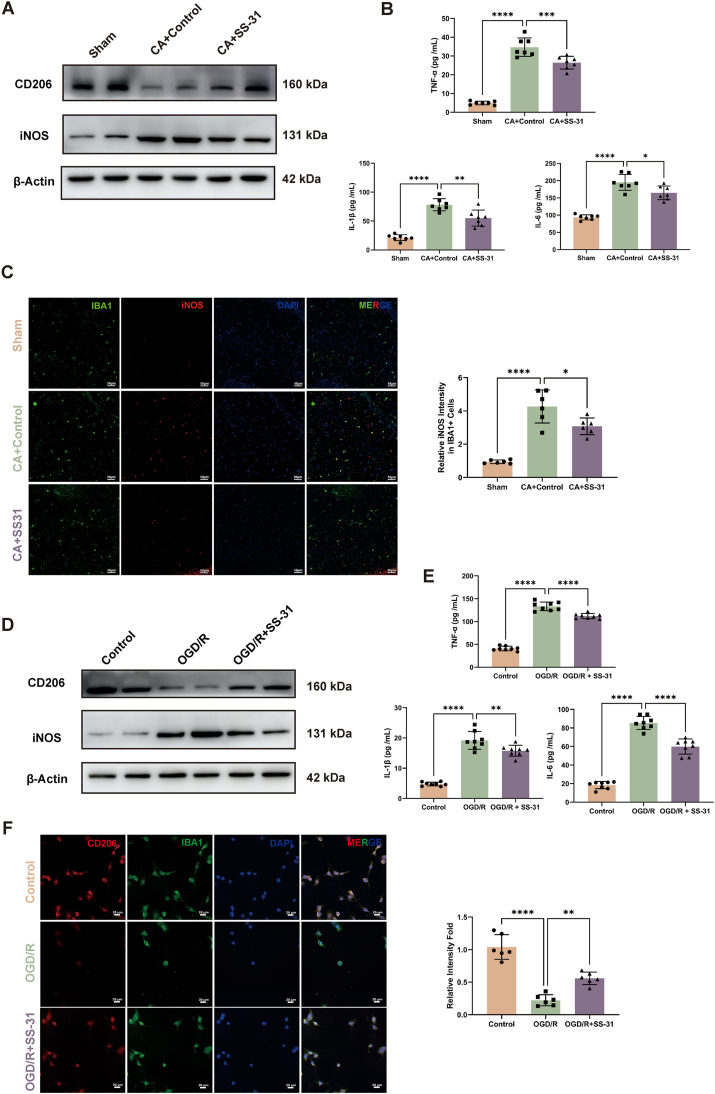
**SS-31 Promotes Microglial M1 to M2 Polarization After Cardiac Arrest and Hypoxia/Reoxygenation**. (A) Western blot analysis of M1 marker INOS and M2 marker CD206 in the hippocampus. (B) Pro-inflammatory cytokine levels (TNF-α, IL-1β, IL-6) in the hippocampus. (C) Immunofluorescence staining of INOS (red) in IBA1+ microglia (green) with DAPI (blue) nuclear staining in the hippocampus. The bar graph shows quantitative analysis of relative iNOS intensity in IBA1+ cells. (D) Western blot analysis of CD206 and iNOS expression in BV2 cells. (E) TNF-α, IL-1β, IL-6 levels in BV2 cells. (F) Immunofluorescence staining of BV2 cells stained for CD206 (red), IBA1 (green), and DAPI (blue). The bar graph shows quantitative analysis of relative CD206 intensity. Statistical significance is indicated as ∗P ​< ​0.05, ∗∗P ​< ​0.01, ∗∗∗P ​< ​0.001, and ∗∗∗∗P ​< ​0.0001. Scale bars are shown in individual panels.

After cerebral ischemia-reperfusion, microglia tend to polarize toward the M1 phenotype, contributing to inflammatory damage. We investigated whether SS-31 could improve microglial polarization and alleviate neuroinflammation resulting from CA. In rat hippocampal tissue, Western blot analysis demonstrated that SS-31 treatment increased the expression of CD206, an M2 microglial marker, and decreased iNOS levels, an M1 marker, after CA (Fig. 5A, Supplementary Fig. 4A). This shift indicates that SS-31 promotes microglial polarization toward the anti-inflammatory M2 phenotype. ELISA analysis revealed that SS-31 treatment significantly reduced pro-inflammatory cytokines TNF-α, IL-1β, and IL-6 in hippocampus in the CA ​+ ​SS-31 group compared to the CA ​+ ​Control group (Fig. 5B). Immunofluorescence staining further confirmed these findings by showing reduced iNOS expression in SS-31-treated groups (Fig. 5C). These results suggest that SS-31 facilitates M1 to M2 polarization of microglia, reducing inflammatory responses and potentially contributing to improved neurological outcomes following CA in vivo.

The therapeutic effects of SS-31 were also validated in vitro. In the BV2 cell, Western blot analysis indicated that SS-31 treatment increased CD206 expression and decreased iNOS levels in the OGD/R model (Fig. 5D, Supplementary Fig. 4B). ELISA analysis showed that SS-31 significantly reduced TNF-α, IL-1β, and IL-6 levels in the OGD/R ​+ ​SS-31 group compared to the OGD/R group (Fig. 5E). Furthermore, immunofluorescence staining demonstrated that OGD/R reduced CD206 expression in BV2, whereas SS-31 treatment restored CD206 levels (Fig. 5F). These results, both in vivo and in vitro, validate the therapeutic effect of SS-31 in promoting microglial M1-to-M2 polarization after CA.

### Sesn2 knockdown in BV2 cells eliminates SS-31's protection against hypoxia-reoxygenation induced ferroptosis

**Fig. 6:**
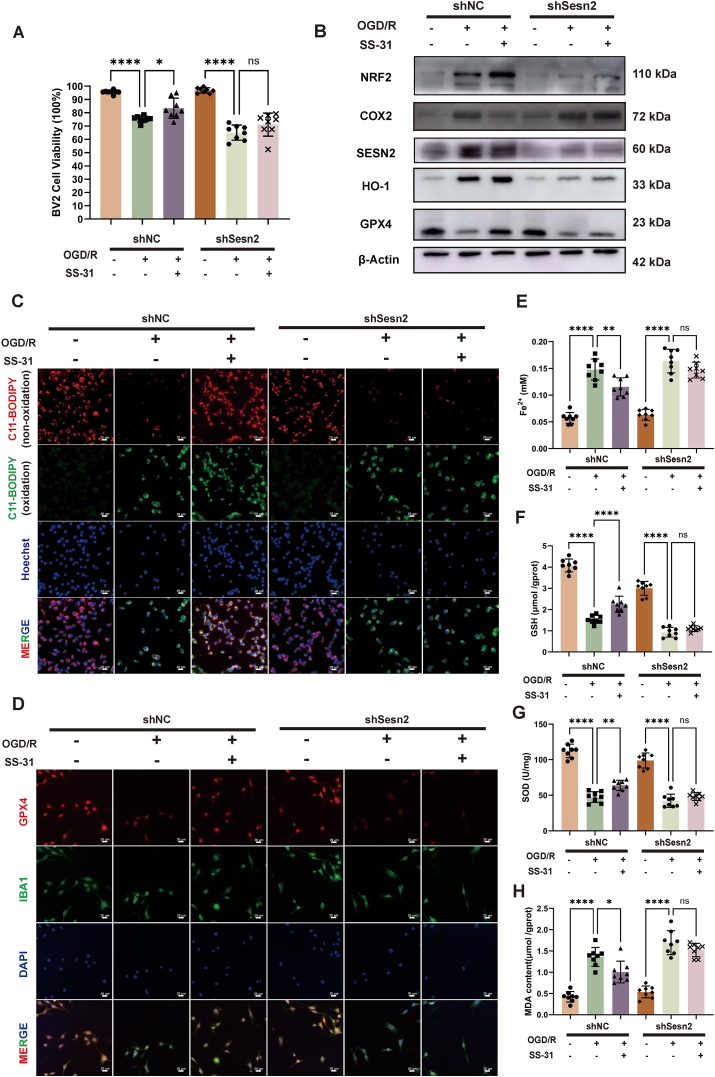
**Sesn2 Knockdown in BV2 Cells Eliminates SS-31's Protection Against Hypoxia-Reoxygenation Induced Ferroptosis**. (A) Cell viability assay (B) Western blot analysis of ferroptosis-related proteins (GPX4, HO-1, NRF2, COX2, SESN2) in BV2 cells. (C) Lipid peroxidation in BV2 cells, as indicated by C11-BODIPY staining (non-oxidized, red; oxidized, green) and nuclear staining with Hoechst (blue). (D) Immunofluorescence staining for GPX4 (red), IBA1 (green), and DAPI (blue) in BV2 cells. (E). Fe^2+^ levels measured in BV2 cells. (F) GSH levels in BV2 cells. (G) SOD levels in BV2 cells. (H) MDA content in BV2 cells. Statistical significance is indicated as follows: ∗P ​< ​0.05, ∗∗P ​< ​0.01, ∗∗∗∗P ​< ​0.0001 and ns ​= ​not significant. Scale bars are shown in individual panels.

Sestrin2 (Sesn2) is a highly conserved, stress-responsive protein that plays a crucial role in protecting against oxidative stress. Sesn2 localizes to mitochondria, where it supports mitochondrial function under stress conditions [24]. Previous studies have shown that Sesn2 protects dendritic cells from sepsis-induced ferroptosis [25]. Additionally, Sesn2 has been reported to provide neuroprotection in ischemic mouse brains by promoting M2 polarization of microglia over M1 [26]. Based on this, we hypothesized that Sesn2 is essential for SS-31's ability to reduce microglial ferroptosis and influence microglial polarization following CA. To assess the role of Sesn2 in SS-31's protective effect in BV2 cells, we used lentiviral transduction to knock down Sesn2 (shSesn2) and compared it with a control knockdown (shNC). SS-31 significantly improved cell viability in the shNC ​+ ​OGD/R group. However, in the shSesn2 ​+ ​OGD/R group, SS-31 did not improve cell viability (Fig. 6A). Western blot analysis showed that in the shNC ​+ ​OGD/R group, SS-31 increased the expression of NRF2, HO-1, and GPX4 while reducing COX2 levels. However, these effects were abolished in the shSesn2 ​+ ​OGD/R group (Fig. 6B, Supplementary Fig. 5A). C11-BODIPY demonstrated that SS-31 reduced lipid peroxidation in the shNC ​+ ​OGD/R group, while this reduction was not observed in the shSesn2 ​+ ​OGD/R group (Fig. 6C, Supplementary Fig. 5B). Additionally, immunofluorescence staining for GPX4 showed increased GPX4 expression with SS-31 treatment in the shNC group, which was not present in the Sesn2 knockdown cells (Fig. 6D, Supplementary Fig. 5C). Furthermore, iron content and GSH, SOD, and MDA, were significantly improved by SS-31 in the shNC group (Fig. 6E–H), but these effects were absent in the shSesn2 group.

### Sesn2 knockdown in microglia eliminates SS-31's protection against CA induced ferroptosis

**Fig. 7:**
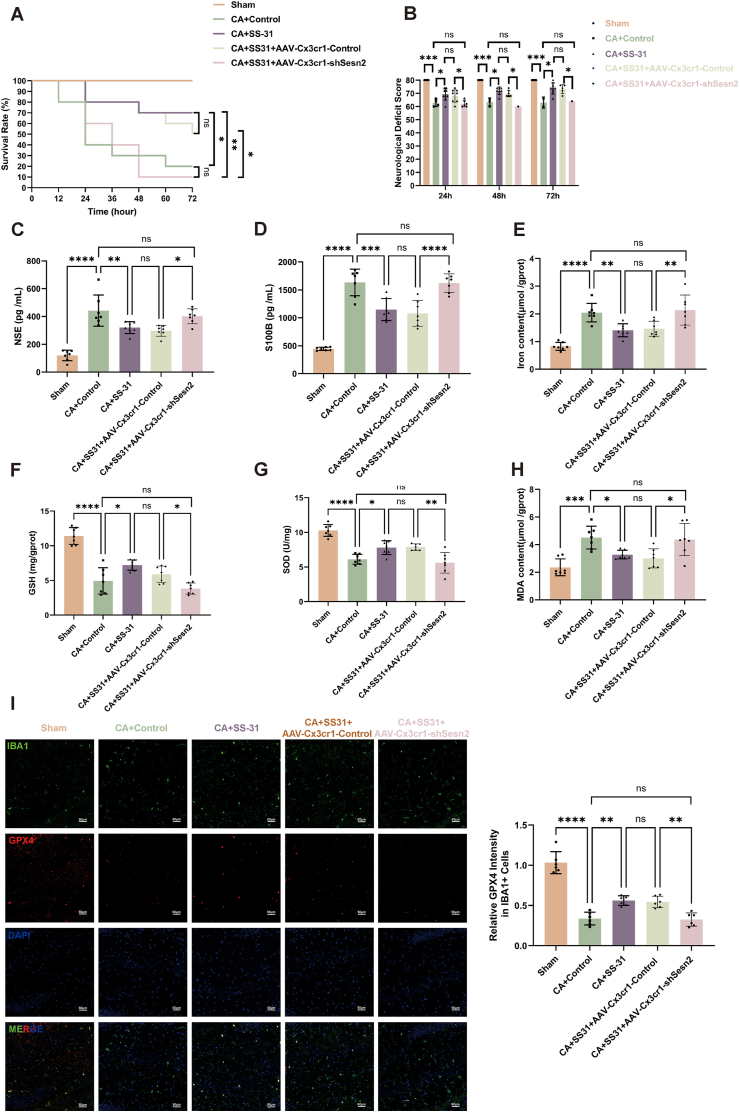
**Sesn2 Knockdown in Microglia Eliminates SS-31's Protection Against Cardiac Arrest Induced Ferroptosis**. (A) Survival analysis comparing groups. (B) Neurological deficit scores at 24, 48, 72 ​h after ROSC. (C) Serum levels of neuron-specific enolase (NSE). (D) Serum levels of S100B. (E) Iron content in the hippocampus. (F) GSH levels in the hippocampus. (G) SOD levels in the hippocampus. (H) MDA content in the hippocampus. (I) Immunofluorescence staining of GPX4 (red) in IBA1+ microglia (green) with DAPI (blue) nuclear staining. The bar graph shows quantitative analysis of relative GPX4 intensity in IBA1+ cells. Statistical significance is indicated as follows: ∗P ​< ​0.05, ∗∗P ​< ​0.01, ∗∗∗P ​< ​0.001, ∗∗∗∗P ​< ​0.0001 and ns ​= ​not significant. Scale bars are shown in individual panels.

To verify the role of Sesn2 in SS-31's protective effect in vivo, we performed stereotactic injection of CA ​+ ​SS-31 ​+ ​AAV-Cx3cr1-shSesn2 virus into the rat hippocampus to specifically knock down Sesn2 in microglia and assess its impact on SS-31's efficacy. Survival analysis showed that, compared to the CA ​+ ​SS-31 ​+ ​AAV-Cx3cr1-Control group, the survival benefit provided by SS-31 treatment was eliminated in the CA ​+ ​SS-31 ​+ ​AAV-Cx3cr1-shSesn2 group at 72 ​h post-CA (Fig. 7A). Additionally, neurological deficit scores indicated that the improvement in neurological function observed with SS-31 treatment was not present in the Sesn2 knockdown group (Fig. 7B). Serum levels of NSE and S100B, markers of neuronal injury, were reduced with SS-31 treatment but remained elevated when Sesn2 was knocked down (Fig. 7C and D).

Furthermore, iron content (Fig. 7E) and MDA levels (Fig. 7H), which were decreased by SS-31 in the CA ​+ ​SS-31 ​+ ​AAV-Cx3cr1-Control group, showed no reduction following Sesn2 knockdown in microglia. Similarly, GSH (Fig. 7F) and SOD (Fig. 7G) levels, which were significantly increased by SS-31, did not improve in the absence of Sesn2. Immunofluorescence staining revealed increased expression of GPX4 in the CA ​+ ​SS-31 ​+ ​AAV-Cx3cr1-Control group. However, this increase was lost in the Sesn2 knockdown group, as evidenced by reduced GPX4 intensity in microglia (Fig. 7I).

### Sesn2 knockdown eliminates the protective effect of SS-31 on microglial M1 to M2 polarization following CA and hypoxia/reoxygenation

**Fig. 8:**
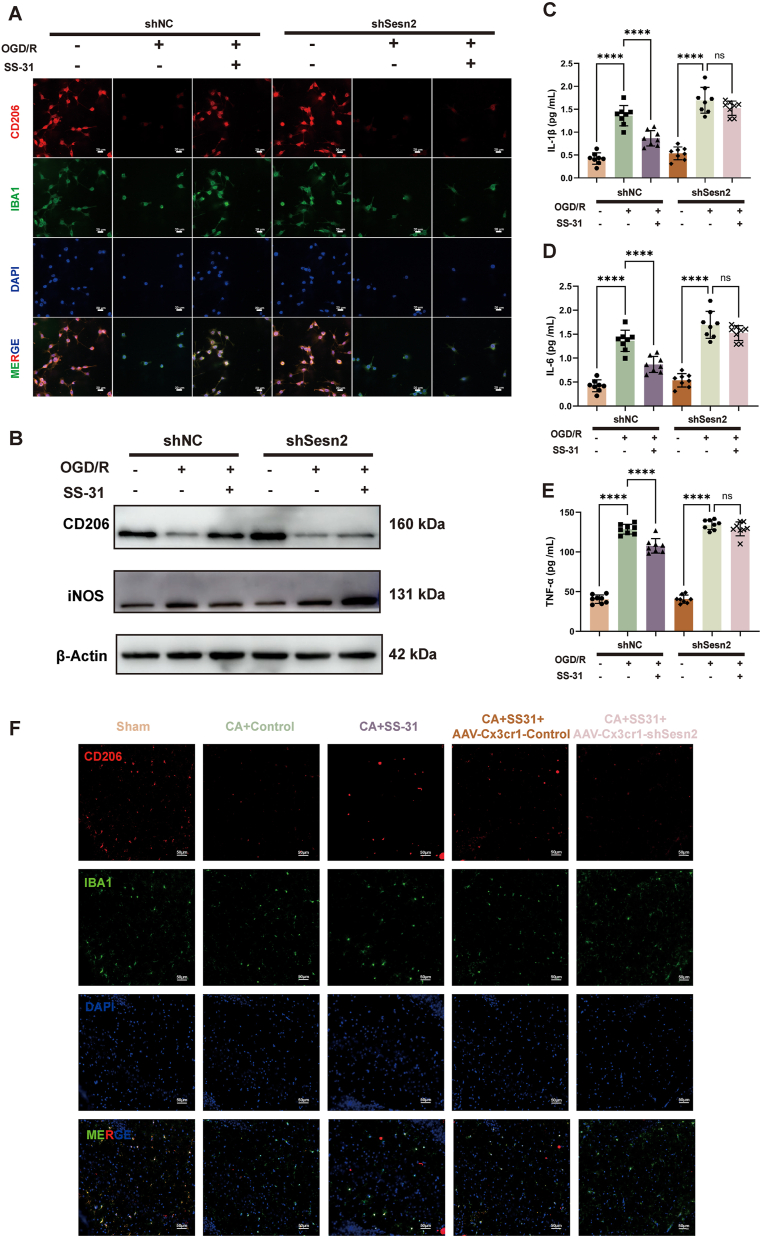
**Sesn2 Knockdown Eliminates the Protective Effect of SS-31 on Microglial M1 to M2 Polarization Following Cardiac Arrest and Hypoxia/Reoxygenation**. (A) Immunofluorescence staining for GPX4 (red), IBA1 (green), and DAPI (blue) in BV2 cells. (B) Western blot analysis of CD206 and iNOS expression in BV2 cells. (C) IL-1β levels. (D) IL-6 levels. (E) TNF-α levels. (F) Immunofluorescence staining of CD206 (red) in IBA1+ microglia (green) with DAPI (blue) nuclear staining. Statistical significance is indicated as follows: ∗∗∗∗P ​< ​0.0001 and ns ​= ​not significant. Scale bars are shown in individual panels.

We further investigated whether the effect of SS-31 in promoting M1-to-M2 polarization of microglia post-CA is mediated by Sesn2. In BV2 cells, immunofluorescence staining showed that SS-31 significantly increased CD206 expression in the shNC group, indicating a shift toward the anti-inflammatory M2 phenotype (Fig. 8A, Supplementary Fig. 6A). This effect was reduced in the shSesn2 group. Western blot analysis confirmed these findings, showing increased CD206 and decreased iNOS levels with SS-31 treatment in the shNC group, but not in the shSesn2 group (Fig. 8B, Supplementary Fig. 6B). Additionally, ELISA results indicated that SS-31 reduced levels of pro-inflammatory cytokines (IL-1β, IL-6, and TNF-α) in the shNC group, while these reductions were absent in the shSesn2 group (Fig. 8C–E).

In the rat hippocampus, after injection of AAV-Cx3cr1-shSesn2, immunofluorescence staining showed that the SS-31-induced increase in CD206 expression in microglia observed in the CA ​+ ​SS-31 ​+ ​AAV-Cx3cr1-Control group was lost (Fig. 8F, Supplementary Fig. 6C). Moreover, the reduction in pro-inflammatory cytokines IL-1β, IL-6, and TNF-α by SS-31 following CA was also abolished after Sesn2 knockdown (Supplementary Fig. 6D).

## Discussion

Current evidence-based treatments, such as extracorporeal membrane oxygenation and targeted temperature management, face significant challenges in widespread clinical application for brain injury following CA due to high resource demands and potential complications [27,28]. Vasopressors and antiarrhythmic drugs used during resuscitation also provide limited benefits for survival and neurological recovery‌ [29]. Therefore, there is an urgent need to develop new neuroprotective drugs to enhance post-CA treatment outcomes. Our key novel findings are: (1) SS-31 significantly improves survival and neurological outcomes by mitigating neuropathological damage; (2) SS-31 exerts protective effects primarily by attenuating mitochondrial oxidative stress, ferroptosis, and excessive M1 polarization in microglia; (3) The beneficial effects of SS-31 on microglial ferroptosis and polarization are mediated through the Sesn2 signaling pathway.

### SS-31: novel modulator of microglial ferroptosis and polarization

Ferroptosis is a recently identified type of regulated cell death distinct from apoptosis and necroptosis, characterized by iron-dependent lipid peroxidation and ROS accumulation [30]. Central regulators of ferroptosis, such as GPX4, SLC7A11, NRF2, HO-1, and COX2, play critical roles in maintaining oxidative balance and iron homeostasis. GPX4, the primary ferroptosis inhibitor, protects cellular membranes from oxidative injury through glutathione (GSH)-dependent lipid peroxide reduction. SLC7A11 supports GPX4 function by facilitating cystine transport into cells, essential for GSH synthesis [31,32]. COX2 expression typically increases during ferroptosis as a marker associated with lipid peroxidation. Previous studies on ferroptosis following CA have not focused specifically on cell-type distinctions [33,34]. Our research specifically targeted microglia due to their pivotal role in neuroinflammation. We observed reduced GPX4 and SLC7A11 expression, increased COX2 expression, mitochondrial abnormalities (shrinkage, disrupted membranes, cristae loss), elevated ROS and MDA levels, increased iron deposition, and decreased antioxidant levels (GSH, SOD) in hippocampal tissues post-CA. These changes confirmed the occurrence of microglial ferroptosis after CA, which was notably reversed by SS-31 treatment.

Iron accumulation also promotes microglial polarization toward the pro-inflammatory M1 phenotype, exacerbating neuroinflammatory responses. Activated microglia release additional iron, ROS, and cellular debris, further activating surrounding microglia and creating a vicious cycle of inflammatory damage [35]. Inhibiting microglial ferroptosis represents a potential therapeutic target for mitigating neuroinflammation. Previous studies have demonstrated that reducing iron accumulation or lipid peroxidation via pharmacological (cepharanthine, resveratrol) or genetic (ACSL4 knockdown, DMT1 inhibition) interventions can effectively attenuate microglial activation, shift polarization toward the M2 anti-inflammatory phenotype, and reduce inflammatory injury [12,36,37]. Consistent with these findings, our study demonstrates that SS-31 effectively suppresses ferroptosis and promotes M2 polarization in both in vitro and in vivo models of CA, significantly reducing neuroinflammation.

### SS-31: multifunctional mitochondrial antioxidant and anti-inflammatory agent

Mitochondria play a crucial role in the pathogenesis of CA, making mitochondrial-targeted therapies a promising approach for CA treatment [14,38]. During CA, cessation of blood flow leads to insufficient energy supply to the brain, impairing the function of energy-dependent ion pumps and disrupting ion homeostasis. This hypoxic depolarization triggers the opening of voltage-gated ion channels, resulting in Ca^2+^ influx and the release of excitatory neurotransmitters like glutamate, which cause excitotoxicity. Excitotoxicity and calcium overload lead to mitochondrial dysfunction, ROS production, and lysosomal activation, ultimately causing cell damage and death, and initiating sterile inflammation [39]. Our study observed mitochondrial damage, increased ROS levels, and elevated inflammatory markers in the hippocampus post-CA. Neuroinflammation, which contributes significantly to secondary brain injury, is mediated by microglia. Previous studies have shown that activated microglia polarize into M1/M2 phenotypes after CA, a finding confirmed by our results [40,41].

SS-31, a novel mitochondrial-targeted antioxidant peptide, can rapidly cross the blood-brain barrier and cell membranes, accumulating in the mitochondrial inner membrane at concentrations more than 5000 times higher than in the cytoplasm [21,42]. Its protective effects have been demonstrated in various animal models of ischemic organ injury [15,[39], [40], [41]]. SS-31 exerts its protective effects through multiple mechanisms, such as binding to cardiolipin on the mitochondrial inner membrane to maintain cristae stability, inhibiting cytochrome *c* peroxidase activity, and reducing electron leakage. Mitochondria are both a major source of ROS production and a primary site for ROS action [43]. Studies have shown that inhibiting mitochondrial ROS, particularly mitochondrial lipid ROS, can reduce ferroptosis [44,45]. Based on the pharmacological mechanism of SS-31, it can decrease mitochondrial ROS production and lipid peroxidation, thereby mitigating ferroptosis, which was confirmed in our findings [15,46]. Additionally, the anti-inflammatory properties of SS-31, demonstrated in various neurological disorders, were also observed in our CA model [47,48].

### SS-31: potential clinical therapeutic agent acting via Sesn2 pathway

The significant efficacy of SS-31 suggests its potential for clinical application, but further studies are needed to explore its pharmacological mechanisms more deeply. After observing that SS-31 improved microglial ferroptosis and polarization, we found through literature review that Sesn2, a protein involved in oxidative stress defense, plays a regulatory role in inhibiting ferroptosis and modulating microglial polarization [25,26,49,50]. Additionally, approximately half of the Sesn2 protein is localized within mitochondria, where it has a direct impact on the regulation of mitochondrial respiratory chain components and provides functional support during mitochondrial stress [24]. Therefore, we hypothesize that the therapeutic effect of SS-31, a mitochondria-specific protective agent, may be mediated through the regulation of the Sesn2 signaling pathway. Sesn2 has been shown to exert protective effects in ischemia-reperfusion injuries in various organs [51,52]. In cerebral ischemia, studies have found that silencing Sesn2 exacerbates cerebral ischemia/reperfusion injury [53]. Sesn2 alleviates focal cerebral ischemic injury by upregulating VEGF expression and promoting angiogenesis [54]. It also promotes neuroprotection by shifting microglial polarization from the M1 to M2 phenotype in ischemic brains [26]. Additionally, Sesn2 can exert antioxidant effects through its downstream target, Nrf2. In Sesn2-silenced conditions, nuclear Nrf2 expression is significantly reduced, whereas Sesn2 overexpression increases nuclear Nrf2 levels and enhances the activation of Nrf2-mediated antioxidant signaling [26,[54], [55], [56]]. Nrf2 is one of the most important transcription factors regulating oxidative stress response. Under stress conditions, it translocates to the nucleus and binds to the antioxidant response element (ARE), initiating the transcription of antioxidant proteins such as HO-1 and glutathione reductase, thereby promoting ROS clearance and reducing oxidative stress [[57], [58], [59]]. Moreover, Nrf2 is a critical transcriptional regulator of anti-ferroptosis, capable of directly or indirectly modulating GPX4 protein levels, which play a key role in reducing lipid peroxidation and free iron accumulation [60,61]. In line with this framework, we observed that the expression levels of Sesn2, Nrf2, and HO-1 were significantly increased in the hippocampal tissue post-CA and were further enhanced by SS-31 treatment. We interpret this pattern as a stress-adaptive response in microglia in which mitochondrial oxidant burden after ischemia–reperfusion induces SESN2 and facilitates NRF2 activity with downstream HO-1 up-regulation, even in the absence of treatment (CA ​+ ​Control), while SS-31 amplifies this adaptation as mitochondrial redox homeostasis is partially restored. At the same time, ferroptotic pressure selectively compromises the cystine–glutathione–GPX4 axis: cystine import via SLC7A11, glutathione availability, and GPX4 function are particularly vulnerable to iron and oxidative stress, which explains why SLC7A11, GSH, and GPX4 decline in CA ​+ ​Control despite activation of SESN2/NRF2/HO-1. By lowering mitochondrial oxidant and iron loads and stabilizing bioenergetics, SS-31 alleviates the constraints that suppress this anti-ferroptotic arm, allowing recovery of SLC7A11, GSH, and GPX4 in concert with sustained SESN2/NRF2/HO-1 activity. Functionally, microglia-specific *Sesn2* knockdown in vivo and shSesn2 in BV2 cells abrogated SS-31–mediated improvements across lipid peroxidation, iron handling, antioxidant indices, cytokine output, neurological scores, and survival, indicating that SESN2 is required for the anti-ferroptotic and anti-inflammatory effects of SS-31 in our models. Notably, while SS-31 treatment was associated with higher SESN2 abundance, our experiments do not establish direct regulation of SESN2 by SS-31. The observed SESN2 increase may reflect an indirect response to restored mitochondrial/redox homeostasis.

### Limitations

First, the CA model was established in healthy rats, which somewhat restricts the direct applicability of the findings to human patients who are typically older and may have a history of cardiovascular disease. Second, detailed cognitive tests (e.g., Morris water maze, open-field test) were not included as study endpoints. Incorporating these tests would provide a more precise quantification of functional differences in learning, memory, and cognitive flexibility across different interventions. Third, at the mechanistic level our data position SESN2 as a required mediator—supported by the abolition of SS-31 benefits after microglial Sesn2 knockdown—yet they do not establish direct regulation of SESN2 by SS-31. Fourth, we did not thoroughly evaluate the mitochondrial protective effects of SS-31 as a mitochondrial-targeted drug. Methods such as measuring mitochondrial oxidative phosphorylation activity, analyzing cardiolipin oxidation products via liquid chromatography-mass spectrometry, and assessing ATP production could offer a more comprehensive understanding of SS-31's impact on mitochondrial function. We will continue to explore the points mentioned above in depth before moving toward clinical translation in the future.

In summary, our findings indicate that SS-31 improves post-CA survival and neurological outcomes in a rat model of CA. The neuroprotective effects of SS-31 are attributed to the suppression of microglial M1 polarization and ferroptosis. Importantly, microglia-specific Sesn2 knockdown abrogated these benefits, positioning SESN2 as a required mediator of SS-31's anti-ferroptotic and anti-inflammatory actions. Using SS-31 as a treatment during CA resuscitation may represent a potential strategy to enhance survival and clinical outcomes in CA patients.

## Consent to publish

Not applicable.

## Author contributions

Tangxing Jiang: Formal analysis, Investigation, Methodology, Project administration, Software, Validation, Writing-original draft. Huidan Zhang: Conceptualization, Formal analysis, Methodology, Software, Writing-review & editing. Yijun Sun: Resources, Writing-review & editing. Xianfei Ji: Supervision, Writing-review & editing. Li Xue: Supervision, Writing-review & editing. Chang Pan: Resources, Supervision, Writing-review & editing. Yunyun Guo: Funding acquisition, Resources, Supervision, Writing-review & editing. Feng Xu: Funding acquisition, Supervision, Writing-review & editing.

## Data availability

Data will be made available on request.

## Ethics approval

This study was approved by the Animal Care and Use Committee of Qilu Hospital, Shandong University. All experimental procedures were conducted in accordance with national and institutional guidelines for the use of laboratory animals. Tissue sampling procedures strictly complied with all relevant institutional and national animal care and welfare regulations.

## Funding

This work was supported by grants from Noncommunicable Chronic Diseases-National Science and Technology Major Project (2023ZD0505504, 2023ZD0505500), the Key R&D Program of Shandong Province (2022ZLGX03), Shandong Provincial Natural Science Foundation (ZR2024QH037).

## Declaration of competing interest

The authors declare that they have no competing interests.

## Abbreviations

AAV: Adeno-Associated Virus
CA: Cardiac arrest
CPR: Cardiopulmonary resuscitation
DHE: Dihydroethidium
ELISA: Enzyme-Linked Immunosorbent Assay
FBS: Fetal Bovine Serum
GPX4: Glutathione Peroxidase 4
HE Staining: Hematoxylin and Eosin Staining
IL-1β: Interleukin-1 beta
IL-6: Interleukin-6
MAP: Mean Arterial Pressure
N/A: Not applicable
NDS: Neurologic Deficit Score
NSE: Neuron-specific enolase
OGD/R: Oxygen-Glucose Deprivation and Reoxygenation
OHCA: Out-of-hospital cardiac arrest
ROSC: Return of spontaneous circulation
ROS: Reactive Oxygen Species
SESN2: Sestrin2
TEM: Transmission Electron Microscopy
TNF-α: Tumor Necrosis Factor-Alpha
