# Supplementary material for: SS-31 improves post-cardiac arrest brain injury by inhibiting microglial ferroptosis and polarization
Source: Neurotherapeutics. 2025 Oct 24;23(1):e00772. doi: 10.1016/j.neurot.2025.e00772 (PMC12976541; doi:10.1016/j.neurot.2025.e00772)
Supplement: Multimedia component 1 [file mmc1.docx]

**Supplementary Materials
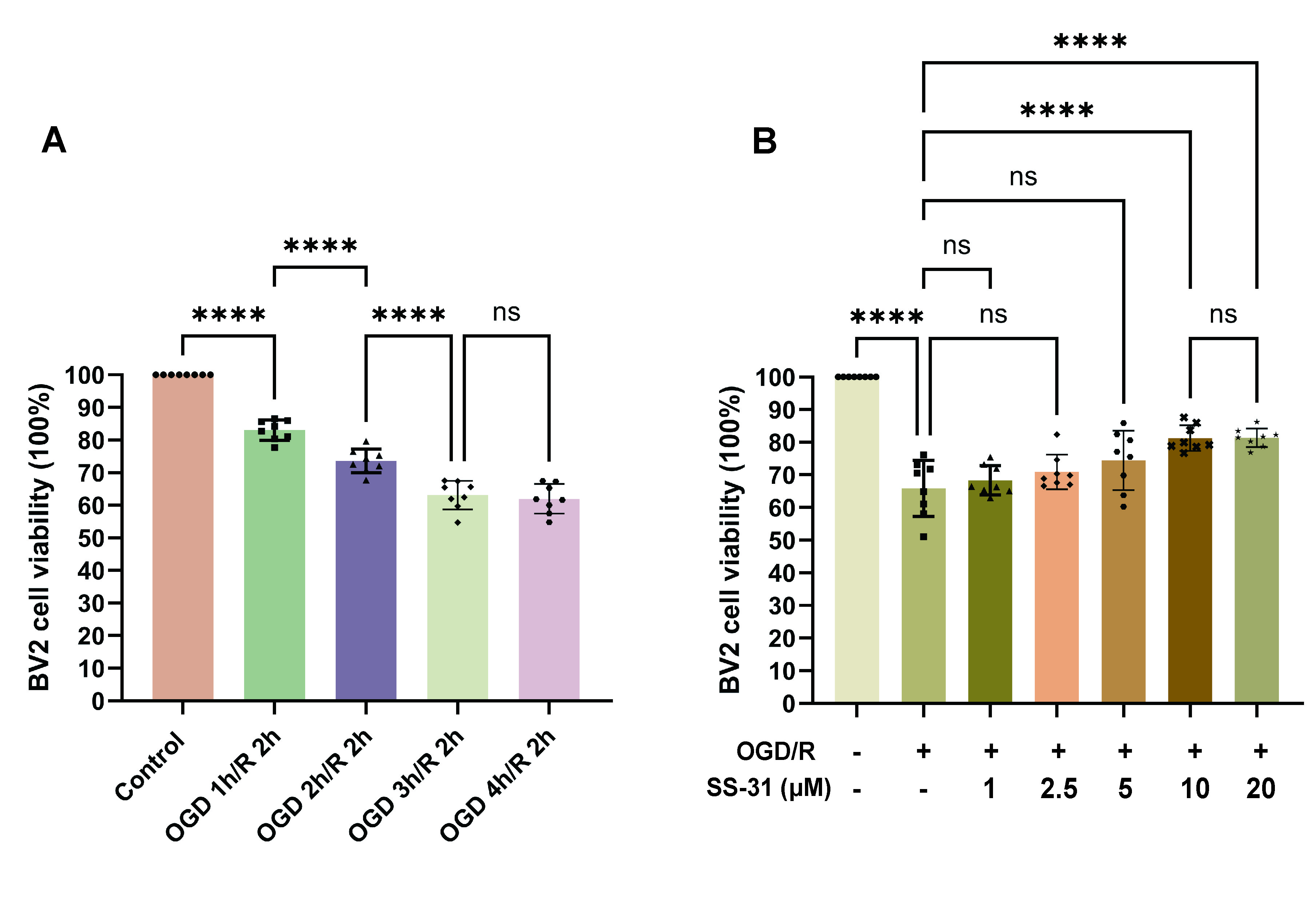
**

**Supplementary Figure 1. Effect of OGD/R Duration and SS-31 Concentration on BV2 Cell Viability**

(A) BV2 cell viability after different durations of OGD/R. (B) Dose-dependent effect of SS-31 on BV2 cell viability after OGD/R. Statistical significance is indicated as follows: ****P < 0.0001, ns = not significant.

**
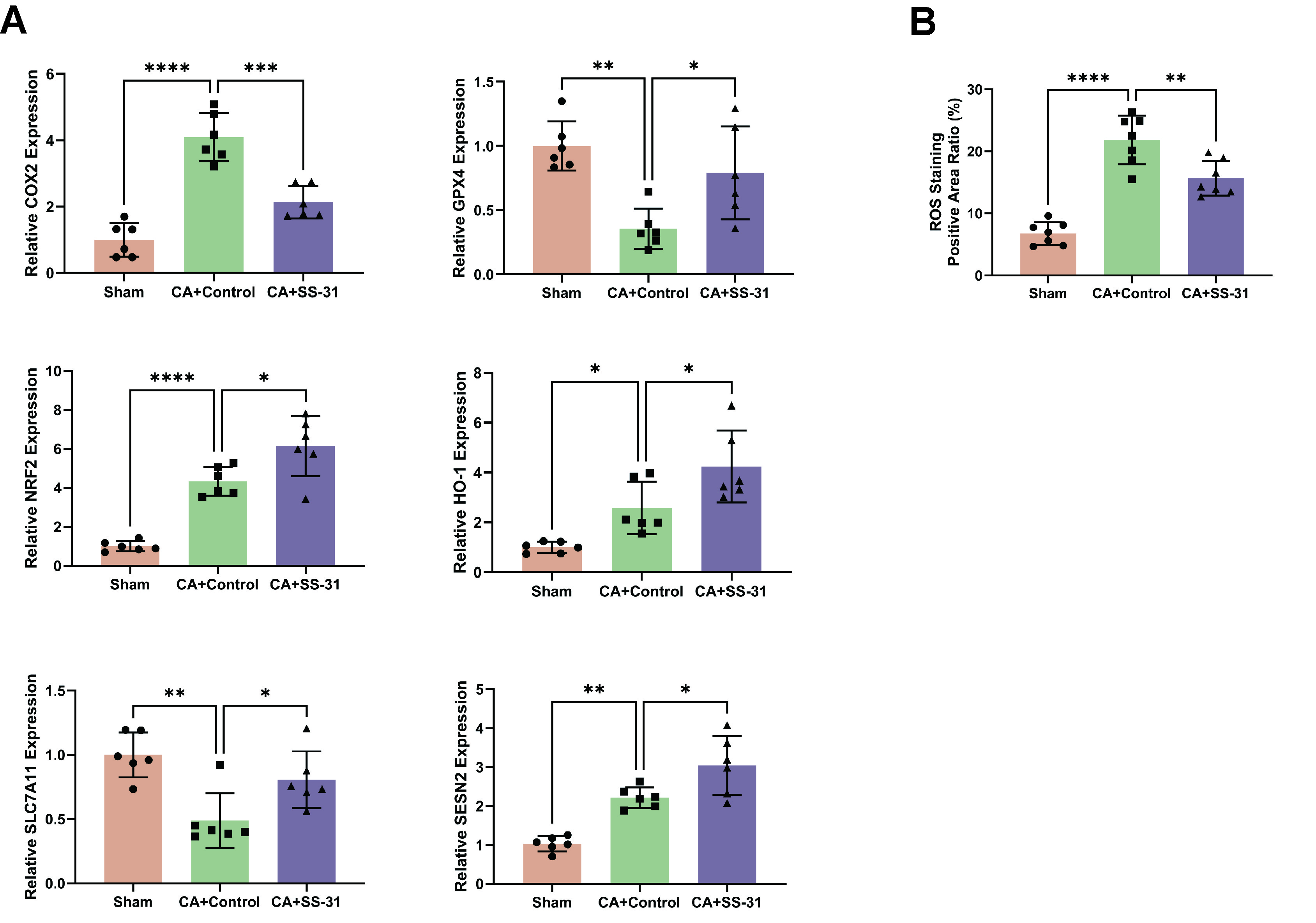
Supplementary Figure 2. SS-31 Reduces Ferroptosis in Hippocampus After Cardiac Arrest**

(A) Quantification of Western blot analysis for GPX4, SLC7A11, HO-1, NRF2, SESN2, and COX2 in the hippocampus. (B) Quantification of ROS staining in hippocampal sections, expressed as a positive area ratio (%). Statistical significance is indicated as follows: *P < 0.05, **P < 0.01, ***P < 0.001, ****P < 0.0001.


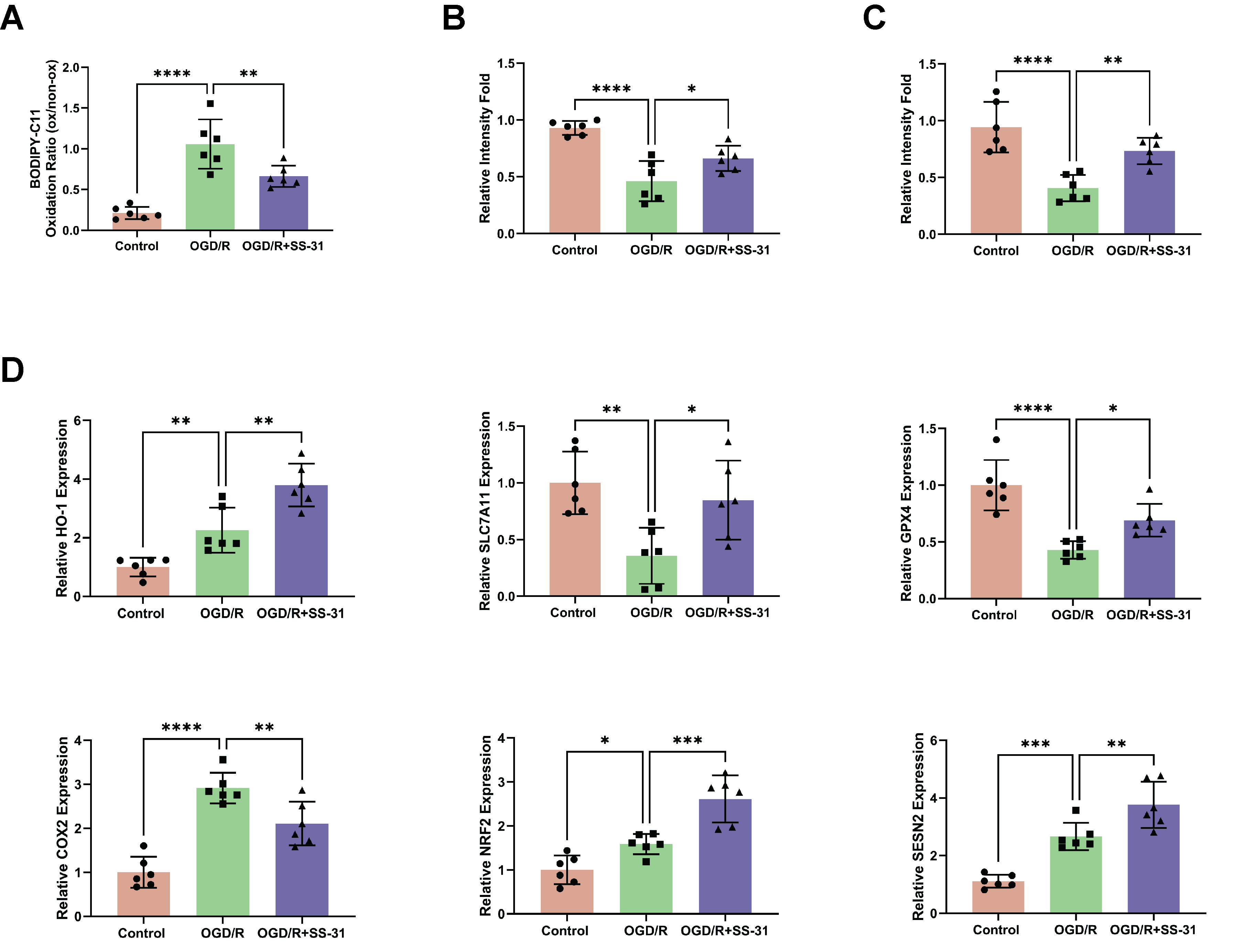


**Supplementary Figure 3. SS-31 Reduces Ferroptosis Induced by Hypoxia-Reoxygenation in BV2 Cells**

(A) Quantification of BODIPY-C11 oxidation ratio (oxidized/non-oxidized) in BV2 cells. (B) Quantitative analysis of SLC7A11 immunofluorescence intensity in BV2 cells. (C) Quantitative analysis of GPX4 immunofluorescence intensity in BV2 cells. (D) Western blot quantification of GPX4, SLC7A11, HO-1, NRF2, COX2, and SESN2. in BV2 cells. Statistical significance is indicated as follows: *P < 0.05, **P < 0.01, ***P < 0.001, ****P < 0.0001.


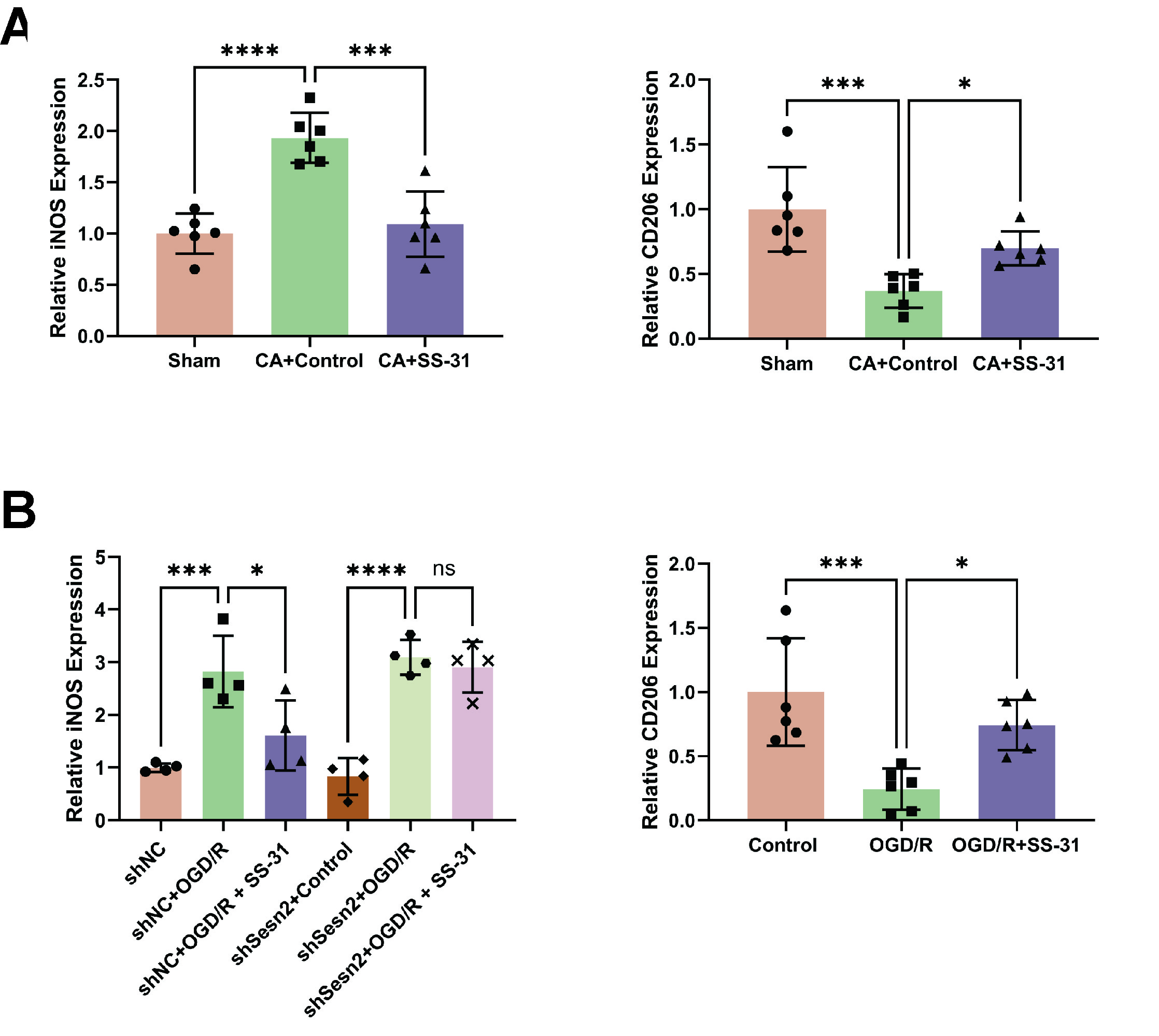


**Supplementary Figure 4. SS-31 Promotes Microglial M1 to M2** **Polarization After Cardiac Arrest and Hypoxia/Reoxygenation**

(A) Western blot analysis quantification of iNOS and CD206 in the hippocampus. (B) Western blot analysis quantification of iNOS and CD206 in BV2 cells. Statistical significance is indicated as follows: *P < 0.05, ***P < 0.001, ****P < 0.0001.


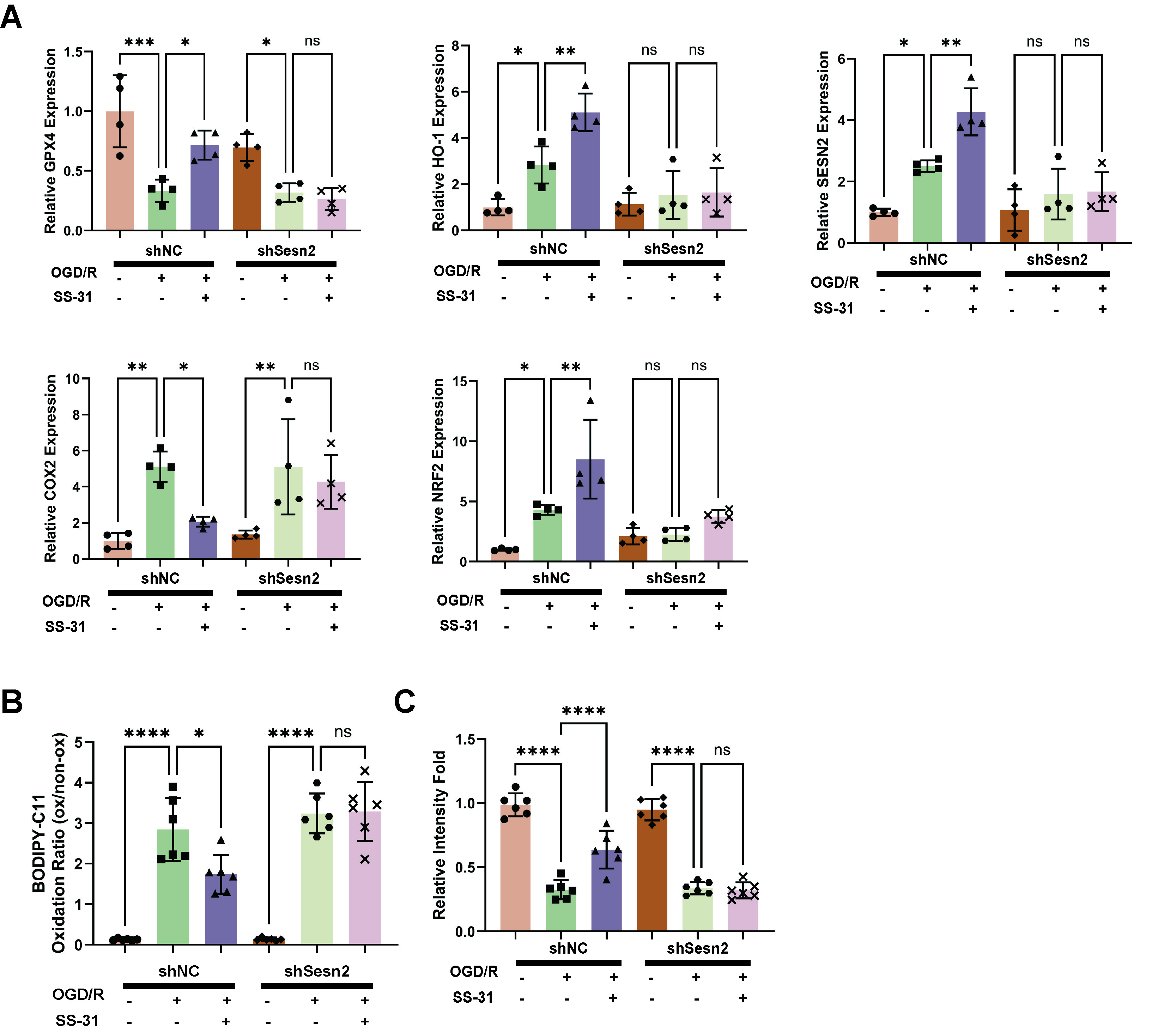


**Supplementary Figure 5. Sesn2 Knockdown in BV2 Cells Eliminates SS-31’s Protection Against Hypoxia-Reoxygenation Induced Ferroptosis**

(A) Western blot analysis quantification of ferroptosis-related protein expression levels, including HO-1, SESN2, GPX4, COX2, and NRF2 in BV2 cells. (B) Quantification of BODIPY-C11 oxidation ratio (oxidized/non-oxidized) in BV2 cells (C) Quantitative analysis of GPX4 immunofluorescence intensity in BV2 cells. Statistical significance is indicated as follows: *P < 0.05, **P < 0.01, ***P < 0.001, ****P < 0.0001.

**
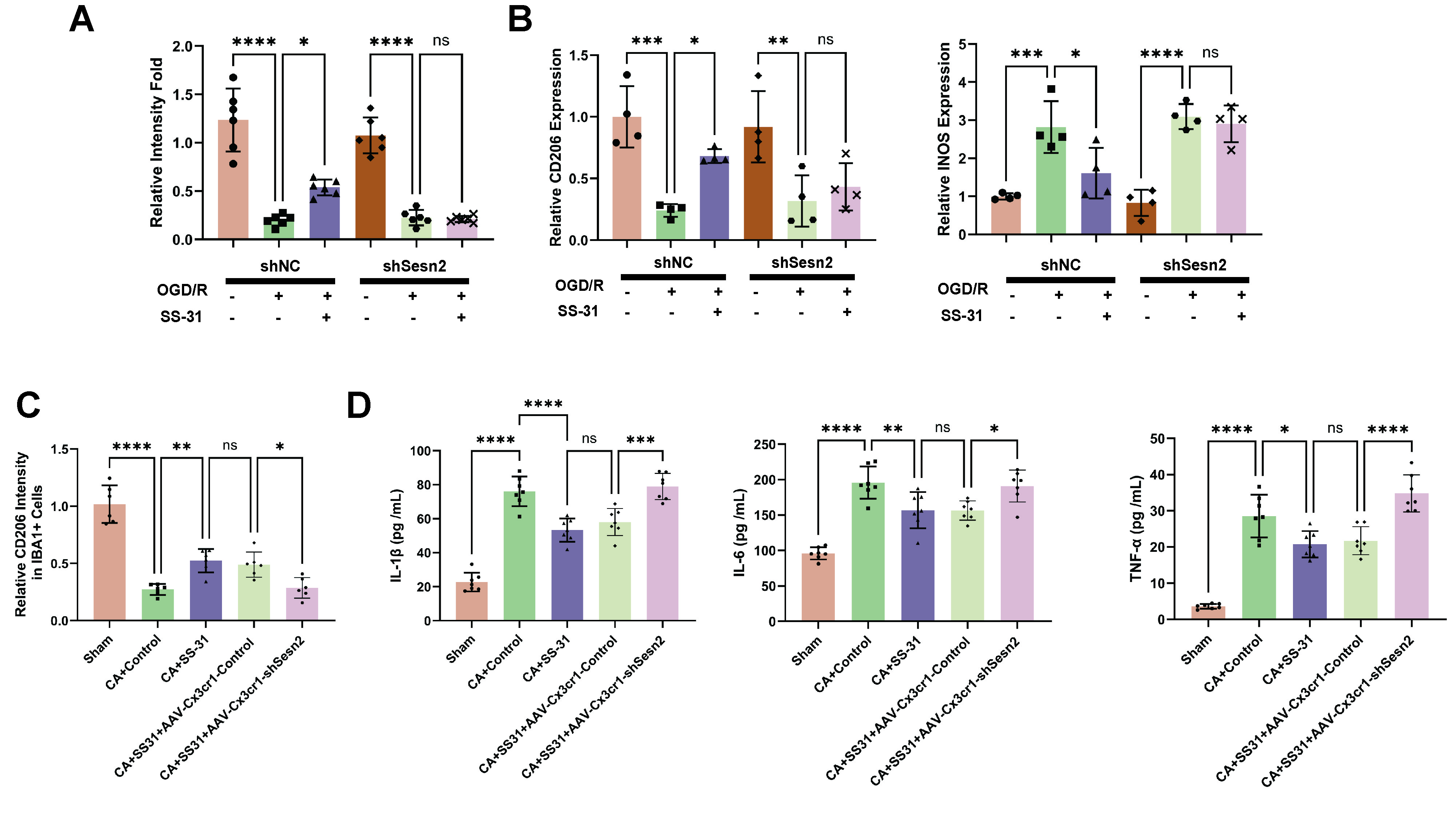
**

**Supplementary Figure 6. Sesn2 Knockdown Eliminates the Protective Effect of SS-31 on Microglial M1 to M2 Polarization Following Cardiac Arrest and Hypoxia/Reoxygenation**

(A) Quantitative analysis of CD206 immunofluorescence intensity in BV2 cells. (B) Western blot analysis quantification of iNOS and CD206 in BV2 cells. (C) Quantification of relative CD206 intensity in IBA1+ cells. (D) Levels of pro-inflammatory cytokines (TNF-α, IL-1β, and IL-6) measured in the hippocampus. Statistical significance is indicated as follows: *P < 0.05, **P < 0.01, ***P < 0.001, ****P < 0.0001 and ns = not significant.

**Supplementary Table 1. NDS Evaluation System (0-80 points)**

| Category | Score Range | Scoring Criteria |
| --- | --- | --- |
| **A. General Behavior Evaluation** |  |  |
| Consciousness and Awareness | 0-10 points | Normal: 10;  Dazed: 5;  No response: 0 |
| Awakening | 0-3 points | Spontaneous awakening: 3; Stimulated awakening: 1;  No awakening: 0 |
| Breathing | 0-6 points | Normal: 6;  Abnormal (too rapid): 3;  No breathing: 0 |
| **B. Brainstem Function** |  |  |
| Swallowing (Response to food intake) | 0-3 points | Present: 3; Absent: 0 |
| Visual (Head movement response to light stimulus) | 0-3 points | Present: 3; Absent: 0 |
| Pupillary Light Reflex | 0-3 points | Present: 3; Absent: 0 |
| Corneal Reflex | 0-3 points | Present: 3; Absent: 0 |
| Startle Reflex | 0-3 points | Present: 3; Absent: 0 |
| Whisker Stimulation Reflex | 0-3 points | Present: 3; Absent: 0 |
| Swallowing: Swallowing solid or liquid food | 0-3 points | Present: 3; Absent: 0 |
| **C. Motor Function Evaluation** |  |  |
| Movement | 0-3 points  (each side) | Normal: 3;  Weak or soft: 1;  No movement or paralysis: 0  (Left and right sides assessed independently) |
| **D. Sensory Function Evaluation** |  |  |
| Response to Pain | 0-3 points  (each side) | Responsive: 3;  Weak response or abnormal (extension, curved posture): 1;  No response: 0 (Left and right sides assessed independently) |
| **E. Autonomous Behavior** |  |  |
| Gait Coordination | 0-3 points | Normal: 3; Abnormal: 1; Absent: 0 |
| Balance Beam Walking | 0-3 points | Normal: 3; Abnormal: 1; Absent: 0 |
| **F. Reflex** |  |  |
| Righting Reflex | 0-3 points | Normal: 3; Abnormal: 1; Absent: 0 |
| Rejection to Elevated Ground Reflex | 0-3 points | Normal: 3; Abnormal: 1; Absent: 0 |
| Visual Coordination | 0-3 points | Normal: 3; Abnormal: 1; Absent: 0 |
| Turning Test | 0-3 points | Normal: 3; Abnormal: 1; Absent: 0 |
| **G. Seizure Activity** |  |  |
| Seizure Activity | 0-10 points | No seizure: 10; Mild seizure: 5; Severe seizure: 0 |

Notes:

1. Scoring Scale: Applicable for rats. Normal score = 80 points, brain death = 0 points.
2. **Balance Beam Walking:** A beam that is 1 cm wide and 0.5 meters long is used. If the rat walks normally across the beam, completing the walk successfully, it is considered normal. If it fails to complete the walk or falls off the beam, it is considered abnormal.
3. **Righting Reflex:** This refers to the righting reflex, where the rat is placed on its back and expected to quickly correct itself into an upright position.
4. **Rejection to Elevated Ground Reflex:** When lifting the rat, if it spreads its limbs and resists being moved beyond 15 cm from the ground, it is considered normal.
5. **Visual Coordination:** Evaluates the rat’s ability to coordinate its vision and movements, ensuring it can adjust its position relative to its surroundings.
6. **Turning Test:** When the rat is tilted at a 45-degree angle, the rat should move its head and body to maintain balance. If the rat cannot balance itself or adjust its position, the test is abnormal.
